# Identification of a Collagen Type I Adhesin of *Bacteroides fragilis*

**DOI:** 10.1371/journal.pone.0091141

**Published:** 2014-03-11

**Authors:** Bruna P. G. V. Galvão, Brandon W. Weber, Mohamed S. Rafudeen, Eliane O. Ferreira, Sheila Patrick, Valerie R. Abratt

**Affiliations:** 1 Department of Molecular and Cell Biology, University of Cape Town, Cape Town, RSA; 2 Structural Biology Research Unit, Division of Medical Biochemistry, Department of Clinical Laboratory Sciences, University of Cape Town, Observatory, Western Cape, South Africa; 3 Departamento de Microbiologia Médica, UFRJ, Instituto de Microbiologia Prof. Paulo de Góes, Ilha do Fundão, Rio de Janeiro, Brazil; 4 Universidade Federal do Rio de Janeiro - Polo Xerém, Duque de Caxias, Rio de Janeiro, Brazil; 5 Centre for Infection and Immunity, School of Medicine, Dentistry and Biomedical Sciences, Queen's University Belfast, Belfast, United Kingdom; Queens University Belfast, Ireland

## Abstract

*Bacteroides fragilis* is an opportunistic pathogen which can cause life threatening infections in humans and animals. The ability to adhere to components of the extracellular matrix, including collagen, is related to bacterial host colonisation. Collagen Far Western analysis of the *B. fragilis* outer membrane protein (OMP) fraction revealed the presence two collagen adhesin bands of ∼31 and ∼34 kDa. The collagen adhesins in the OMP fraction were separated and isolated by two-dimensional SDS-PAGE and also purified by collagen affinity chromatography. The collagen binding proteins isolated by both these independent methods were subjected to tandem mass spectroscopy for peptide identification and matched to a single hypothetical protein encoded by *B. fragilis* NCTC 9343 (BF0586), conserved in YCH46 (BF0662) and 638R (BF0633) and which is designated in this study as *cbp1* (collagen binding protein). Functionality of the protein was confirmed by targeted insertional mutagenesis of the *cbp1* gene in *B. fragilis* GSH18 which resulted in the specific loss of both the ∼31 kDa and the ∼34 kDa adhesin bands. Purified his-tagged Cbp1, expressed in a *B. fragilis* wild-type and a glycosylation deficient mutant, confirmed that the *cbp1* gene encoded the observed collagen adhesin, and showed that the 34 kDa band represents a glycosylated version of the ∼31 kDa protein. Glycosylation did not appear to be required for binding collagen. This study is the first to report the presence of collagen type I adhesin proteins in *B. fragilis* and to functionally identify a gene encoding a collagen binding protein.

## Introduction

*Bacteroides fragilis*, a Gram negative, rod-shaped obligately anaerobic bacterium, is a commensal inhabitant of the human colon, assisting with digestion and the development of the host immune system [1]. However, under certain circumstances, it can become an opportunistic pathogen causing abscess formation, soft-tissue infections, and bacteraemia [2]. Treatment of these life-threatening infections is complicated by the emergence of antibiotic drug resistance in the clinical setting [3] and, therefore, alternative novel therapeutic targets, such as factors which enhance bacterial virulence, need to be investigated to assist in drug design.

Potential *B. fragilis* virulence factors include capsular polysaccharides, neuraminidases (sialidases), haemolysins and enterotoxins (*B. fragilis* toxin; BFT) [2]. When a breach in the epithelium of the human intestine occurs, *B. fragilis* comes into contact with the host extracellular matrix (ECM). The ability of the bacterium to interact with components of the ECM is thought to play a major role in pathogenesis involving both adhesion to and degradation of these components during colonization and tissue invasion. The host ECM is an intricate network of biologically active macromolecules underlying the epithelial and endothelial cells. It surrounds connective tissue cells serving a structural function and participating in cellular adhesion, migration, proliferation and differentiation [4]. The ECM also serves as a substrate for the attachment of microorganisms [5], a crucial first step in the infective process. This attachment is mediated by microbial surface components which can recognise adhesive matrix molecules (MSCRAMMs) [5]. Inhibition of the adhesive properties of organisms has been examined as a potential therapeutic method, and strategies for preventing adhesion have been reviewed by Barczak and Hung [6]. For example, in Gram negative bacteria, the inhibition of the formation of pili and fimbriae has been shown to block adhesion [7]. The macromolecules that make up the ECM are divided into four main classes, namely glycoproteins, proteoglycans, elastin and collagens. *B. fragilis* has been shown to interact with a number of these components including the glycoproteins laminin and fibronectin, and the proteoglycan fibrinogen [8], [9], [10], [11].

Collagen is the major component of the ECM and is the most abundant protein in mammals, accounting for 25 to 33% of all proteins [12]. The ability of bacteria to interact with it is, therefore, important for both commensals and pathogens. Adhesins with the ability to bind single or multiple forms of collagen have been described in bacteria [5]. The Cna, collagen binding protein mediates the adhesion of *Staphylococcus aureus* to different collagen types [13], and the virulence factor YadA, found in pathogenic *Yersinia* spp. [14], binds to various collagen types (I, III, IV and V) as well as the ECM proteins laminin and fibronectin.

There is limited published information relating to the interactions of *B. fragilis* with collagen. Szoke *et al*. [15] demonstrated collagen type I binding associated with cell surface proteinaceous receptors. Fifteen of 24 *B. fragilis* isolates from infected sites and 9 out of the 13 of strains isolated from faecal material were able to adhere to collagen type I [10] indicating that adhesion was strain specific. This was supported by the work of Galvão *et al*. [16] who showed that 87% of the *B. fragilis* clinical isolates studied could bind collagen. In that study, *B. fragilis* strain GSH18 demonstrated the highest level of collagen binding. We now report the functional characterisation of a glycoprotein collagen adhesin from *B. fragilis* GSH18.

## Materials and Methods

### Bacterial Strains, Plasmids and Growth Conditions

Bacterial strains and plasmids are shown in Table 1. *B. fragilis* was routinely cultured in supplemented Difco brain heart infusion medium (BHIS), and grown under anaerobic conditions [17]. *B. fragilis* transconjugants were cultured on BHIS agar including gentamicin (200 µg/ml) and erythromycin (10 µg/ml). *B. fragilis* strains carrying the plasmid pCMF92 were grown in the presence of erythromycin (10 µg/ml). *Escherichia coli* strains were grown, aerobically, in Luria-Bertani (LB) broth or agar at 37°C [18] and supplemented with ampicillin (100 µg/µl) to maintain plasmids.

**Table 1:** Description of bacterial strains and plasmids.

Strain/Plasmid	Genotype/phenotype^a^	Source/reference
**Plasmids**		
**pGERM**	pUC19-based suicide vector	[28]
**pGERMcbp1-int**	pGERM containing *cbp1* internal fragment	This study
**pCMF92**	pCMF6 expressing Cbp1 with a C-terminal His-tag	[34]
**Strains**		
***Bacteroides fragilis***		
**GSH18**	Clinical isolate, Gent^R^, Tet^R^	[16]
**GSH18 ** ***cbp1^−^***	GSH18 derivative, *cbp1^−^*, Gent^R^Erm^R^	This study
**NCTC9343 pCMF92**	Wildtype strain expressing Cbp1^His^; Erm^R^	[34]
**Δgmd-fclΔfkp pCMF92**	Glycosylation deficient mutant expressing Cbp1^His^; Erm^R^	[34]
***Escherichia coli***		
**S17-1**	RP4-2-Tc::Mu *aph*::Tn7*recA^−^*, Strep^R^	[37]
**S17-1 pGERMcbp1-int**	RP4-2-Tc::Mu *aph*::Tn7*recA^−^*, Strep^R^Amp^R^	This study

Gent^R^, Erm^R^, Strep^R^, and Amp^R^ denote resistance to gentamicin, erythromycin, streptomycin and ampicillin, respectively

### Membrane Protein Sample Preparation

*B. fragilis* GSH18 cells grown for 16 h to late log/early stationary phase, were collected and disrupted by sonication using a Misonic sonicator 3000 at a power output of 3 W for 5 rounds of 30 s. The outer membrane proteins (OMP) were then extracted by the method of the Pauer *et al*. [9], [19]. OMPs were resuspended in 1 X PBS (pH 7.4). For the purification of the His-tagged proteins (see later), crude membrane (CM) samples were used. These were obtained before the 0.3% Sarcosyl treatment step of the OMP extraction technique. The protein concentrations were determined using Bio-Rad Protein Assay Dye Reagent Concentrate according to the manufacturer's instructions (Bio-Rad Laboratories, cat # 1500-0006) using bovine serum albumin (BSA) as a standard.

### Collagen Far Western Blot

An adaption of the methods of Esmay *et al*. [20] and Ferreira *et al*. [8] was used. The OMP sample (75 µg) was mixed in a 1∶1 ratio with 2X SDS gel loading buffer (0.125 M Tris-HCl [pH 6.8], 4% SDS, 20% glycerol, 0.002% bromophenol blue) and incubated at room temperature for 10 min prior to electrophoresis on duplicate gels (10% SDS (w/v) polyacrylamide gel without β-mercaptoethanol) [21]. PageRulerTM Prestained Protein Ladder (Fermentas, #SM0671) was used as the molecular weight standard. One gel was stained with Coomassie brilliant blue or Acqua stain (Acqua Science), and the other was transferred on to a nitrocellulose membrane using the Trans-Blot® SD DNA/RNA Blotting Kit (Bio-Rad) as per the manufacturer's instructions. A minimum of three biological repeats were performed for each experiment.

The nitrocellulose blots were blocked overnight in blocking buffer (1X PBS buffer, pH 7.2 with 1.5% BSA [Roche, 10 735 086 001] and 5% skim milk) followed by 3 h incubation at approximately 23°C in PBS-B-T buffer (1X PBS, pH 7.2; 1.5% BSA; 0.1% Tween) containing 20 µg/ml of collagen Type I from calf skin (Elastin Products Company Inc.). The membranes were immunostained with anti-collagen type I (Rabbit) antibody (Rockland, 600-401-103-0.1) and peroxidase conjugated anti-rabbit IgG (Goat) antibody (Rockland, 611-1302; or Gene Tex, GTX77060) as primary and secondary antibodies, respectively. TMB Membrane Peroxidase substrate (KPL, 50-77–18) or Pierce ECL Western Blotting Chemiluminescent Substrate (Thermo Scientific Product # 32106) was used for development of the membranes. In order to confirm the specificity of the anti-collagen type I (Rabbit) antibody a control blot was exposed to the antibody without the collagen binding step.

### Collagen Affinity Chromatography

Collagen affinity purification of collagen binding OMP proteins was performed using a cyanogen bromide (CnBr) activated sepharose matrix (Sigma, C9142) coupled with collagen Type I prepared as per the manufacturer's instructions. The OMP fraction of *B. fragilis* GSH18 (3-7 µg in 10 ml 1 X PBS) was passed three times through the collagen affinity column which was then washed with 20 ml 1 X PBS. The bound proteins were eluted in a step-wise manner with 2 ml of 0.15, 0.3, 0.6, 1, 1.5, and 2 M NaCl solutions. The protein content of 200 µl of the OMP sample, and 2 ml of each elution fraction were precipitated using 100% (w/v) Trichloroacetic acid (TCA) [22]. Each protein pellet was resuspended in 20 µl of 1X SDS-PAGE loading dye and examined on SDS –polyacrylamide gel (10% (w/v) without β-mercaptoethanol.

### Two-dimensional (2D) Gel Electrophoresis

OMPs (250 µg) were TCA precipitated by the method of Isaacson *et al*. [22]. Solubilisation and sample application on to the ReadyStrip™ IPG strips (BIORAD) were done according to the manufacturer's instructions. Iso-electric focusing (IEF) was performed in a Protean IEF Cell (Bio-Rad) as follows: 250 V, 15 m; 2500 V, 2 h30 m; 4000 V, 20 000 Vh. The second dimension was done on a 10% SDS polyacrylamide gel. Four replicate gels were run for analysis and protein spot picking. Duplicate gels were run for Far Western analysis.

### LC MS/MS Analysis of Putative Adhesins

Protein bands excised from the affinity purification gels and protein spots excised from the 2-D PAGE gels were analyzed by tandem mass spectrometry (LC MS/MS) (Yale Cancer Centre Mass Spectrometry Resource & W.M. Keck Foundation Biotechnology Resource Laboratory, New Haven, Connecticut, USA). The proteins were identified against the NCBI bacterial database using the MASCOT program (www.matrixscience.com), where protein scores greater than 82 were significant (*p*<0.05).

### Bioinformatic analysis of the *cbp1* gene cluster

Bioinformatic analysis of the predicted protein sequence of the *B. fragilis* NCTC 9343, *cbp1* gene (BF0586) was performed using Phyre2 (Protein Homology/AnalogY Recognition Engine), an automatic fold recognition server for predicting the structure and/or function of protein sequences [23]. Clustal Omega was used to generate the multiple sequence alignments [24], [25]. The predicted protein sequence of the *cbp1* gene product was analysed using the LipoP 1.0 and GlycoPP servers [26], [27].

### DNA Techniques and Construction of *B. fragilis cbp1-* mutant

Genomic DNA of *B. fragilis* GSH18 was extracted using the method of Steffens *et al*. [17]. Plasmids were extracted from *E. coli* strains using the BioSpin Plasmid Extraction Kit (BioFlux). All PCR analyses were performed using Kapa ReadyMix (Inqaba Biotech) as per manufacturers' instructions, and the thermal cycling was performed using a GeneAmp® PCR system 9700 (Applied Biosystems). The standard PCR parameters used consisted of an initial 5 min denaturation step at 95°C, followed by 25 cycles of denaturation for 30 sec at 95°C, annealing for 30 sec at primer pair specific temperatures (Table 2), and elongation at 72°C for time periods specific to target products, followed by a final 5 min elongation step at 72°C.

**Table 2:** Description of PCR primers.

Name	Primer	*Hyb T°	Fragment size	Description
**BF0586-Fstop**	5′-GCT**TAG**TGTAACGGCCTGG-3′	52.1°C	0.339 kb	Primer pair amplifies an internal fragment of BF0586 of *B. fragilis* NCTC 9343 gene homologue; *cbp1*. Stop codons are bolded with altered bp underlined.
				
**BF0586-Rstop**	5′-ACTCAACAGCCTT**CTA**CAGC-3′	50.4°C		
				
**M13F**	5′-CGCCAGGGTTTTCCCAGTCACGAC-3′	60.5°C	variable	Universal primers [38]
				
**M13R**	5′-GAGCGGATAACAATTTCACACAGG-3′	53.7°C		
				
**BF0586-F2PET**	5′-AAATGTAAT**CCCATGG**AAAAGTTAGC-3′ (*Nco*I)	51.0°C	0.87 kb	Primer pair amplifies the full length BF0586 of *B. fragilis* NCTC 9343 gene homologue; *cbp1*. Restriction enzyme sites (in brackets) are bolded with altered bp underlined.
				
**BF0586-R2PET**	5′-TTA**CTCGAG**TTGGATAACCAAATAC-3′ (*Xho*I)	50.7°C		
				

*Hyb T°**.** Hybridization temperature.

For construction of the *B. fragilis* GSH18 *cbp1* ^−^ insertional mutant, a 0.339 kb fragment corresponding to an internal region of the *cbp1* gene, beginning at base 43 and terminating at base 381 was PCR amplified using the primers BF0586-Fstop and BF0586-Rstop (Table 2). The product was blunt cloned into the *Sma*I site of the suicide vector pGERM according to standard protocols [18] to generate pGERMcbp1-int. The recombinant plasmid was transformed into *E. coli* S17-1 competent cells and transferred into *B. fragilis* GSH18 by conjugation [28]. Confirmation of mutagenesis was done by PCR of erythromycin and gentamicin resistant *B. fragilis* transconjugant colonies, using primers M13R with BF0586-F2PET, and M13F with BF0586-R2PET (Table 2), followed by sequencing of the PCR products (Macrogen Inc., Seoul, Korea).

### Purification of His-tagged Proteins Expressed in Wild-Type *B. fragilis* 9343 and its Glycosylation Deficient Mutant (Δgmd-fclΔfkp)

The His-tagged proteins expressed in each strain were purified from the respective crude membrane extracts (CMs) as follows. A 5 ml Hitrap chelating column (GE Healthcare) was charged with nickel and equilibrated with 10 column volumes of buffer A (0.1 M sodium phosphate buffer, pH 8.0, 500 mM NaCl, 8 M urea). The protein sample was filtered through a 0.45 µm filter and loaded onto the column. Once the OD_280_ returned to baseline the column was washed with buffer B (0.1 M sodium phosphate, pH 6.3, 500 mM NaCl, 8 M urea). The bound protein was eluted using a linear gradient of 15 column volumes to 50% buffer C (0.1 M sodium phosphate, pH 6.3, 500 mM NaCl, 8 M urea, 500 mM Imidazole) and a step to 100% buffer C which was maintained for 5 column volumes.

### Whole cell collagen adhesion assays

The collagen adhesion assays were performed based on a previously described method [8]. Briefly, 20 µg/ml of collagen Type I from calf skin (Elastin Products Company Inc.) in PBS was immobilized on glass cover slips for 1 hour. The cover slips were subsequently washed with PBS containing 0.1% (w/v) Bovine Serum albumin (BSA) (0.1% PBSB) to remove unbound collagen. Finally, the cover slips were blocked with 2% PBSB for another hour. Cover slips coated with 2% BSA were used as a control. Suspensions of either the *B. fragilis* GSH18 wild-type, or the *cbp1* ^−^ mutant, at an OD_600 nm_ of approximately 0.6 were prepared using pre-reduced PBS. Aliquots (200 µl) of each strain were added to the cover slips and allowed to interact anaerobically for 60 min at 37°C. After the incubation period, the cover slips were washed with PBS and fixed with methanol for 5 min, and then washed again with PBS. The bound bacterial cells were then stained with 0.1% crystal violet. Each experiment was carried out in triplicate (3 biological replicates) with 3 technical repeats each. The adherence of the bacteria to the cover slips was determined by counting 10 random fields of view of each cover slip (1000× magnification). Data are presented as the mean ±SEM (Standard Error of the mean). The Student t-test was used for statistical analysis and values with *p*<0.05 were considered significant.

## Results and Discussion

### Collagen Adhesins of the *B. fragilis* Outer Membrane

**Figure 1:**
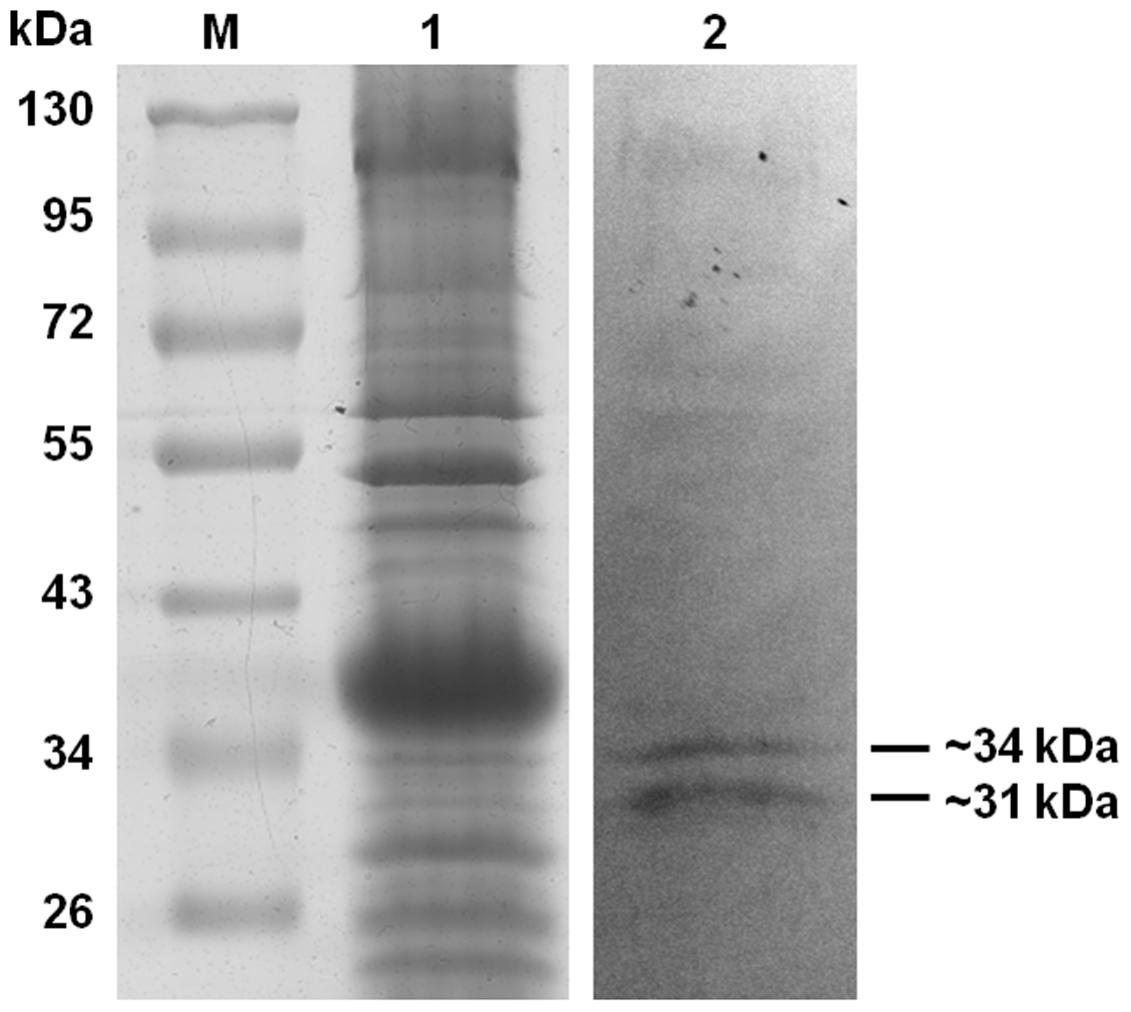
Collagen Far Western of the outer membrane protein fraction (OMP) of *B. fragilis* GSH18. Lanes : (**M**): PageRuler™ #SM0671; (**1**) Coomassie stained gel, (**2**) Far Western.

**Figure 2:**
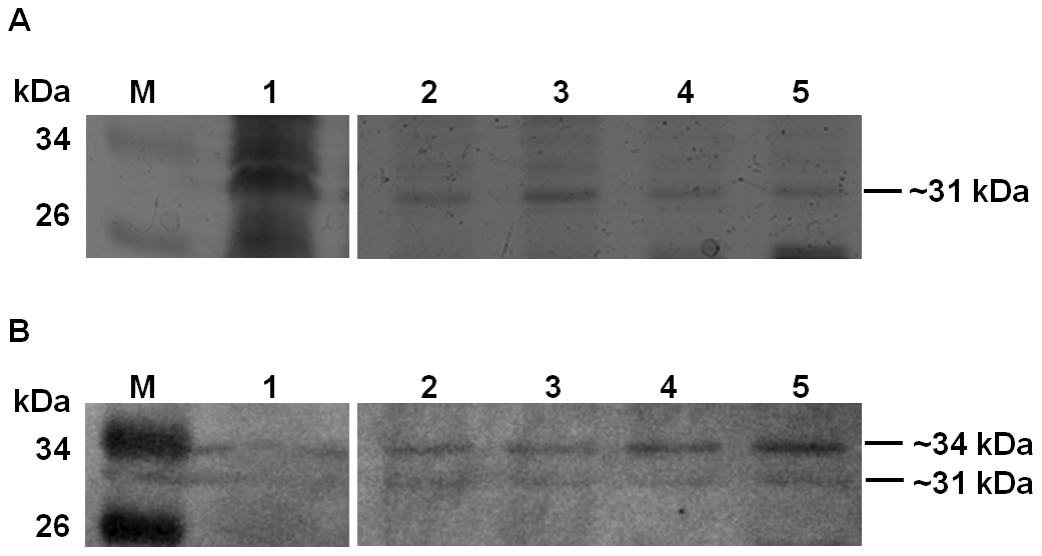
Affinity purification of collagen adhesins of the outer membrane protein fraction (OMP) of *B. fragilis* GSH18: (**A**) Coomassie stained gel, (**B**) Far Western. **Lanes**: (**M**): PageRuler™ #SM0671, (**1**): OMP, (**2–5**): Elutions with 0.6, 1, 1.5 and 2 M NaCl respectively.

The ability of *B. fragilis* whole cells to adhere to collagen has been previously demonstrated by us and others [16], [15], [10], but the cell surface structures responsible for this adhesion were not known. In order to identify such proteins, the collagen adhesion profile of the outer membrane proteins (OMPs) of the *B. fragilis* clinical isolate GSH18 was examined by collagen Far Western analysis (Lane 2, Fig. 1). This showed the production of putative collagen adhesins of approximately ∼31 and ∼34 kDa. Two independent methods were used in parallel to isolate and separate these. The first method, affinity chromatography, allowed for the enrichment of the putative adhesins (Fig. 2A). Although the presence of both the ∼31 and the ∼34 kDa putative adhesins could be clearly seen in the Far Western analysis of the eluted fractions (Fig. 2B, lanes 2 to 5) only a ∼31 kDa protein band appeared more intensely in the coomassie stained gel (Fig. 2A, lanes 2 to 5). This band eluted best using 1 M NaCl solution (Fig. 2A, lane 3) and was excised from the protein gel and subjected to LC MS/MS analysis.

**Figure 3:**
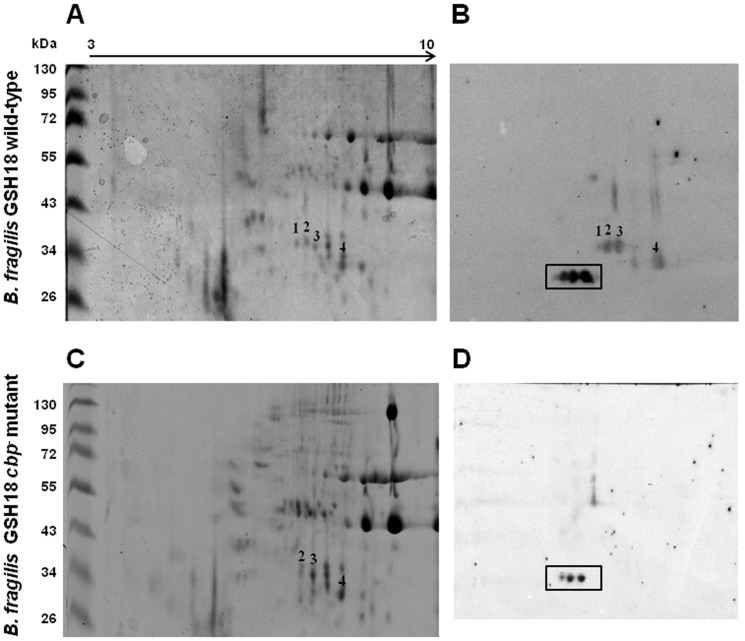
Comparison of collagen-binding proteins from the outer membrane of *B. fragilis* GSH18 wild-type and *B. fragilis* GSH18 *cbp1* ^−^ mutant by two-dimensional gel electrophoresis. OMPs were separated in the first dimension on precast IPG strips (pH 3–10, indicated by the arrow) followed by separation in the second dimension on 10% SDS-PAGE gels: (**A and C**) Coomassie stained gels of the wild-type and *cbp1* ^−^ respectively; (**B and D**) Collagen Far Western of proteins from 2D-gels of the wild-type and *cbp1* ^-^ respectively;. **M**: PageRuler™ #SM0671. Further details discussed in text.

The second method used to isolate putative collagen binding OMPs was 2D-PAGE. The aim here was to separate out all the proteins of the OMP fraction, identify those which could bind collagen and then excise single protein spots for protein identification. In order to observe the full spectrum of possible OMP adhesins in the 2D-PAGE experiments, a pI range of 3 to 10 was used. Figures 3A and B show an example of one of the 2D-PAGE gels and the corresponding Far Western blots. The Coomassie stained gels were analyzed, using the PDQuest Software version 7.4.0 (Bio-Rad), in order to match particular protein spots across the various gels. A number of protein spots able to bind collagen were observed. These were predominantly found in an area between approximately pI 5.7 and 7.3, with a size range between 25 and 54 kDa. A very intense area of collagen binding could be seen at approximately 28 kDa (indicated by the box in Fig. 3B). Unfortunately we were not able to clearly see corresponding spots in the Coomassie stained gels (Fig. 3A) and these proteins could not be analysed further using this method. It is possible that these proteins have been post-translationally modified to such an extent that renders them recalcitrant to staining by Coomassie. Future studies to identify these proteins could make use of commercially available fluorescent dyes such as Flamingo Pink™ (BioRad) and Sypro Ruby™ (Invitrogen), which are more sensitive than Coomassie, and can stain even glycosylated proteins efficiently, and require little or no pre-treatment before being subjected to mass spectrometric analysis. Of the other collagen binding spots observed, those labelled 1 to 4 in Figures 3B appeared to correspond to coomassie stained spots labelled in the same manner. These spots were in the correct size range for the bands seen in the 1D gels and were, therefore, excised from the protein gels and subjected to LC MS/MS analysis.

### Identification of a *B. fragilis* collagen adhesin protein

The LC MS/MS analysis of the affinity purified ∼31 kDa sample showed the highest scoring candidate to be a hypothetical protein, BF0586, of *B. fragilis* NCTC9343. Interestingly protein spots 1, 2 and 3, isolated independently in the 2D-PAGE experiment, also had this hypothetical protein as the highest scoring candidate. Spot 4 had this protein only as its third highest scoring candidate, with its highest scoring candidate being a putative succinate dehydrogenase/fumarate reductase iron-sulfur subunit (BF4341, of *B. fragilis* NCTC9343). The BF0586 gene, to be referred to from this point onwards as *cbp1* (**c**ollagen **b**inding **p**rotein), is predicted to encode a protein with a calculated molecular mass of 31.6 kDa. This is in the correct size range for the smaller of the putative adhesin bands (Fig 1) and for the 2D spots observed in this study (Fig 3A). This gene was, therefore, explored further as a possible candidate for encoding this observed collagen binding protein.

This gene is conserved in the genomes of the three published *B. fragilis* genome sequences. The BF0586 gene of *B. fragilis* NCTC 9343 [29] corresponds to the YCH46 [30] ORF BF0662, and ORF BF638R0633 of strain 638R [31]. The *cbp1* gene is annotated in the genome (NCBI) as encoding a 31.6 kDa hypothetical protein with a predicted Smc, chromosome segregation domain (e-value of 2.20e-07) and no homology to any adhesin-like proteins. Attempts to further analyse the predicted protein sequence of the putative Cbp1 protein using the automatic fold recognition server Phyre2 did not yield any further useful information.

**Figure 4:**
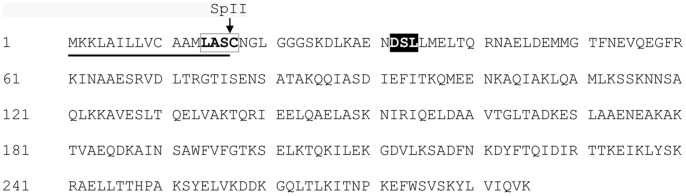
Predicted protein sequence of the pre-protein version of Cbp1 with the putative post-translation modification sites and regions of interest indicated. A signal peptide was predicted by LipoP 1.0 to be found from amino acid position 1 to 16 (underlined) with a putative signal peptidase type II (SpII) site between S16 and C17. A characteristic lipobox consensus sequence, [**L**VI][**A**STVI][GA**S**]C, is indicated by the boxed amino acids in bold font with C17 being the predicted conserved site of acylation. The sequence shaded in black and in white font indicates the *B. fragilis* O-glycosylation motif(s) **D**(**S**/T)(A/I/**L**/M/T/V).

The LipoP 1.0 server indicated that Cbp1 is a putative lipoprotein (Fig 4). The presence of a signal sequence, as well as a lipobox motif strongly supports this prediction [32]. The signal sequence is predicted to be found from amino acid position 1 to 16 with a putative signal peptidase type II (SpII) site between S16 and C17. A characteristic lipobox consensus sequence, [**L**VI][**A**STVI][GA**S**]C, is present with C17 being the predicted conserved site of acylation. According to Kovacs-Simon and colleagues [32] the thiol group of this cysteine residue of lipoproteins receives a diacylglyceryl moiety which known to anchor the proteins to the membrane. In Gram negative bacteria such as *B. fragilis*, the mature lipoprotein also has an additional fatty acid that is linked to the N-terminus of the same cysteine residue. Acylation is not only used to tether proteins to the bacterial cell membranes but is also important for virulence. Lipoproteins are involved in host-pathogen interactions which range from initiation of inflammation to cell surface adhesion. These characteristics are relevant for the proposed role of Cbp1 as a cell surface adhesin and are consistent with our observation that this protein is found in the membrane fraction of *B. fragilis*.

Glycosylation is another post-translational modification (PTM) that has been shown to be an important feature of proteins involved in pathogenesis. These include cell surface molecules such as adhesins and the protein subunits of pili, structures known to be involved in adhesion in *B. fragilis* [33], [2]. Several mutants of bacterial genes responsible for the glycosylation of proteins have been shown to have reduced adhesion ability [33]. The recently developed GlycoPP 1.0 server is an updated tool for the prediction of glycosites of prokaryotic glycoproteins which incorporates the work of Fletcher and colleagues on the characteristics of O-glycosylation in *B. fragilis* [27], [34]. These sources were use to detect the putative O-glycosylation sites D32, S33 and L34 of Cbp1 which are consistent with *B. fragilis* O-glycosylation motif(s) **D**(**S**/T)(A/I/**L**/M/T/V). Interestingly Fletcher and colleagues identified Cbp1 (BF0586) as a putative glycoprotein using this bioinformatics prediction tool, and also determined that the protein is glycosylated using immuno-detection with anti-glycan antiserum. In their study, however, they suggest a putative role for the protein in chromosomal segregation.

### Insertional inactivation of *cbp1* and functional confirmation

In order to test whether Cbp1 (BF0586) was responsible for the observed collagen adhesion seen in *B. fragilis* by Far Western analysis, insertional mutagenesis was used to disrupt the gene encoding it. The disruption was confirmed by PCR of the transconjugant strain with primers external to the gene (BF0586-F2PET and BF0586-R2PET) in combination with M13 primers specific to the suicide vector used (Table 2), and the resultant products were sequenced (data not shown).

**Figure 5:**
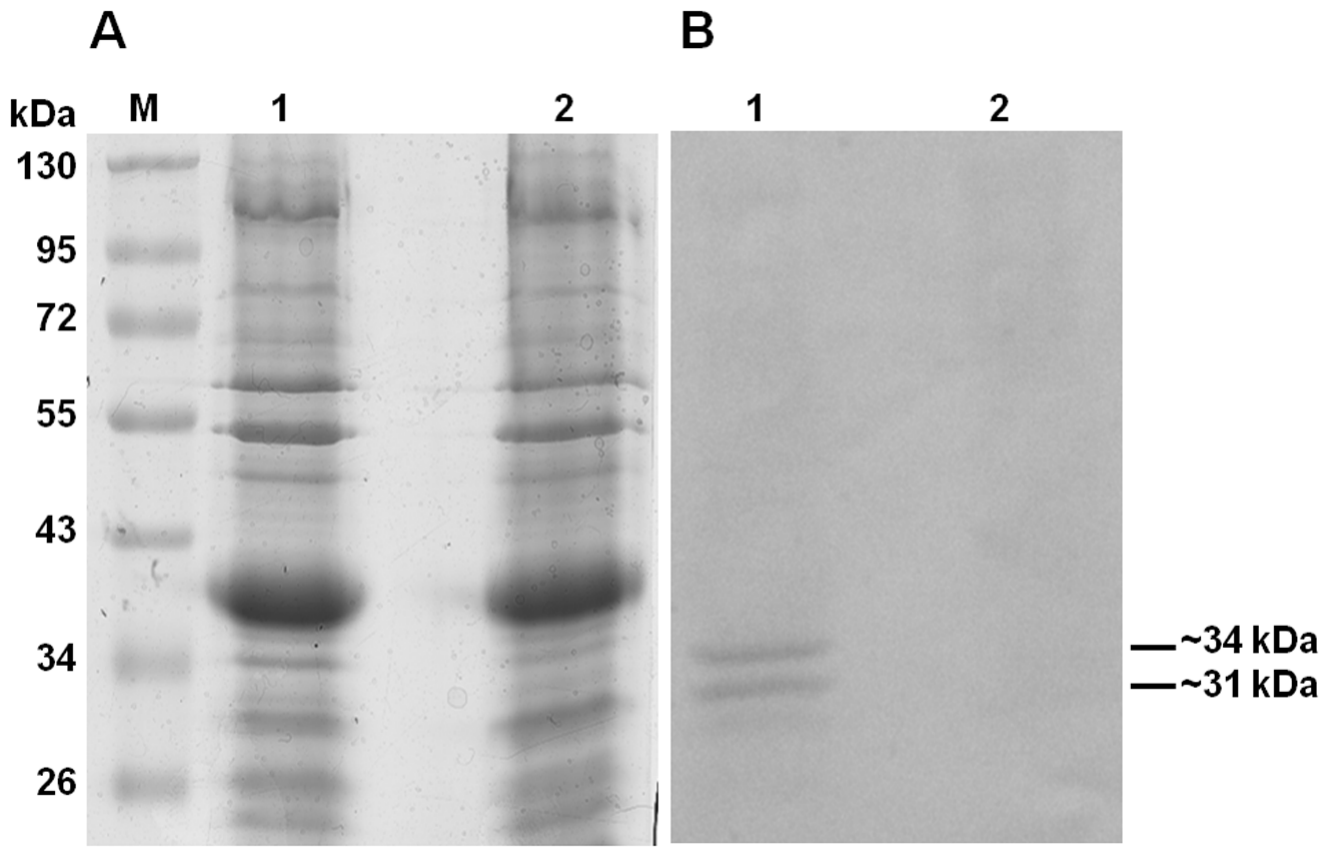
Collagen Far Western blot of *B. fragilis* GSH18 wild-type and *cbp1* ^−^ mutant. (**A**) Coomassie stained gel, (**B**) Far Western. **Lanes**: (**M**): PageRuler™ #SM0671; (**1**) wild-type OMP; (**2**) *cbp1* ^-^mutant OMP.

A comparison of the collagen adhesin profile of the OMPs of *B. fragilis* GSH18 *cbp1* ^−^ mutant, with that of the wild-type strain, was performed using both one- and two-dimensional (1D and 2D) gel electrophoresis (Figures 5 and 3 respectively). The collagen Far Western analysis of these revealed that the *B. fragilis cbp1* mutant lacked both the ∼31 and ∼34 kDa collagen binding bands on the 1D gel (lane 2 of Figure 5 B) as well as all 4 spots previously chosen for analysis from the 2D gels (spots 1 to 4 of Figure 3 D). It was not possible to see a distinct difference between the *B. fragilis* wild-type and *cbp1^−^* mutant in the coomassie stained protein gels of the 1D protein gels, however, an analysis of 2D gels using the PDQuest Software version 7.4.0 (Bio-Rad) showed that only spot 1, was clearly absent in the mutant (Fig. 3C). It remains unclear why spots 2 to 4 on the coomassie gels were still present in the mutant but it is possible that in the wild-type, differentially modified forms of Cbp1 may have co-migrated with other proteins also present in the same position as these spots.

These results show that the product of the *cbp1* gene is responsible for the production of the two collagen binding protein bands observed in the 1D- Far Western blots. The size of the smaller band (∼31 kDa) agrees with the predicted protein size of 31.6 kDa (unmodified pre-protein) and the predicted size of 29.98 kDa of the protein with the cleaved signal sequence. The variation of sizes and pI's observed for the Cbp1 in the 1D- and 2D analyses may, therefore, be a reflection of the presence of Cbp1 in various stages of post-translational modification, including the removal of the signal sequence, and the addition of the predicted lipid and sugar moieties discussed previously. The work of Fletcher and colleagues [34] showed that the glycosylated forms of the *B. fragilis* proteins studied all showed an increase in apparent molecular weight. Interestingly all the variations of the protein observed in the present study bound collagen so it was not clear if glycosylation itself influenced this function. To validate that the *cbp1* gene is responsible for the production of the ∼31 and ∼34 kDa collagen binding proteins that are produced by the wild type and not by the *cbp1^−^* mutant strains, a His-tagged form of Cbp1 (Cbp1^His^) was purified and subjected to collagen Far Western analysis.

### Binding of Collagen by the purified Cbp1^His^

**Figure 6:**
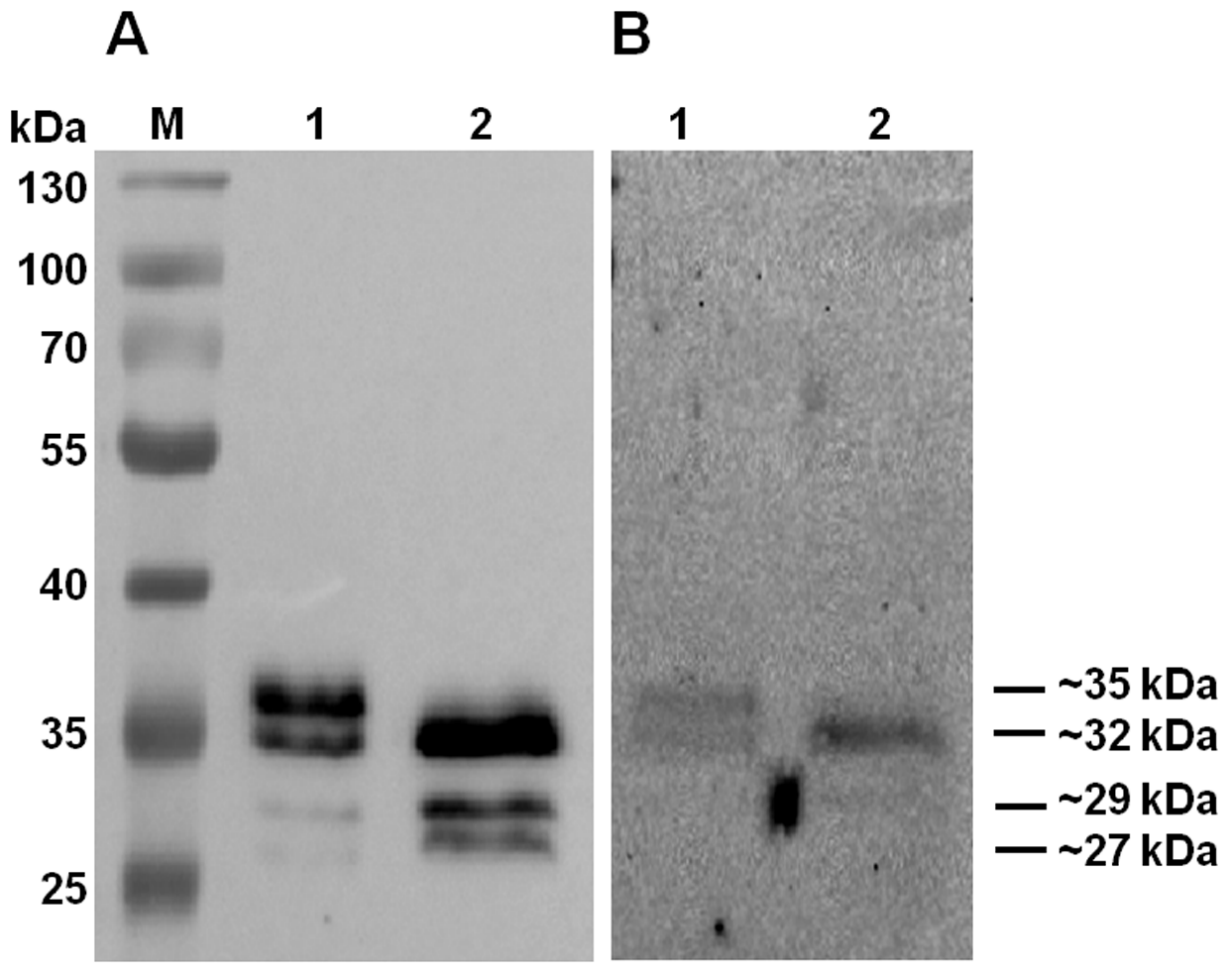
Analysis of the purified Cbp1^His^ proteins produced from wildtype *B. fragilis* 9343 and the glycosylation deficient mutant *B. fragilis* 9343 (Δgmd-fclΔfkp). (**A**) His-Tag Western (**B**) Collagen Far Western. **Lanes**: (**M**): PageRuler™ #SM0671; (**1**) Cbp1^His^ from wildtype *B. fragilis* NCTC 9343; (**2**) Cbp1^His^ from *B. fragilis* Δgmd-fclΔfkp.

The target proteins were expressed in the *B. fragilis* hosts to ensure that any relevant post-translational modifications (PTMs) could be completed. This is important since heterologous expression in *E. coli* is not always effective in achieving the same PTMs as the native host [35]. The Cbp1^His^ was purified by nickel affinity chromatography from extracts from both wild-type NCTC 9343 (WT Cbp1^His^) and the glycosylation deficient mutant, Δgmd-fclΔfkp (ΔΔ Cbp1^His^) [34]. Figure 6A shows that the purified Cbp1^His^ is present chiefly as a ∼35 and ∼32 kDa band in the WT Cbp1^His^ and as a ∼32 kDa band in the ΔΔ Cbp1^His^. The ∼35 kDa band can, therefore, be interpreted to represent the glycosylated form of the protein. Both forms of the protein can, however, bind collagen (Fig. 6B). This result supports the data shown in Figure 5B where the mutation in the *cbp1* gene results in the loss of both bands.

It is important to note that two smaller his-tagged protein fragments of ∼27 and ∼29 kDa (Fig 6A) were co-purified with the target proteins that during the nickel purification process. All the His-tagged proteins were analysed by LC MS/MS and it was revealed that the fragments smaller than ∼32 kDa were truncated versions of the Cbp1^His^ protein. The truncated fragments were shown to lack the N-terminal regions down to about position R73 (Fig 4), whereas the ∼32 and ∼35 kDa extended to about position A29. This would account for the presence of the C-terminal his-tag observed in the smaller fragments (Fig 6A) and would explain why these fragments were co-purified with the target proteins. The reason for the occurrence of the truncated fragments is not clear. The apparent lack of collagen adhesion by the fragments may be due to the species being present at low concentrations. Alternatively, the collagen binding domain could be located at the N-terminal end of the protein. Future investigations will determine which regions of the protein are essential for collagen binding, by analysing collagen adhesion using N-terminal truncated versions of Cbp1.

### Whole cell collagen adhesion assays

**Figure 7:**
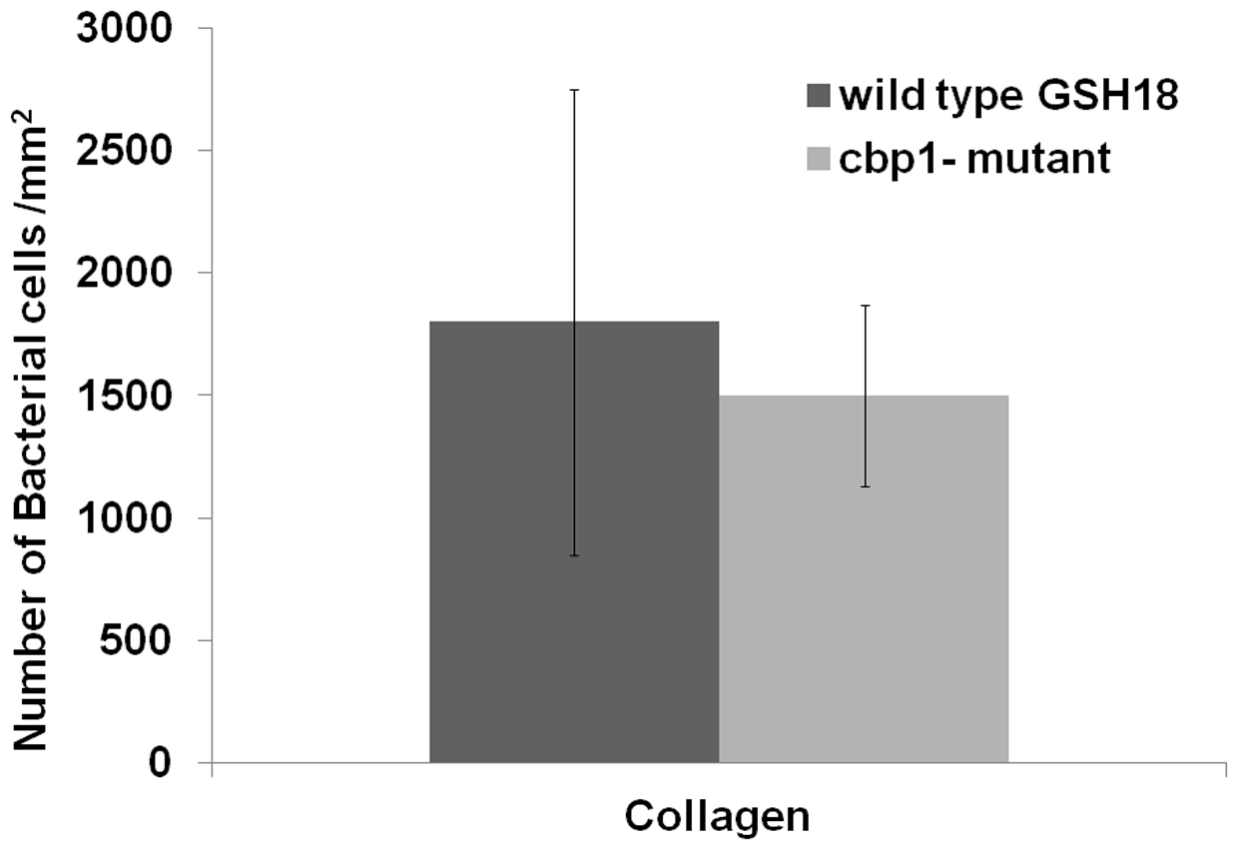
Adhesion of *B. fragilis* GSH18 wild-type and *cbp1* ^-^ mutant to collagen. Experiments performed in triplicate. Data are presented as the mean ±SEM (Standard Error of the mean). The Student t-test was used for statistical analysis and values with *p*<0.05 were considered significant.

To analyze the contribution of Cbp1 to the ability of whole cells of *B. fragilis* to adhere to collagen type I *in vitro*, the *B. fragilis* GSH 18 wild-type and *cbp1* ^−^ mutant strains were allowed to adhere to collagen type I immobilized on glass coverslips. The number of bound bacterial cells was then determined. This revealed that there was no significant difference between the ability of the whole cells of *B. fragilis* GSH 18 wild-type and *cbp1* ^−^ mutant to bind collagen (*p*-value = 0.464) (Fig. 7). This indicates that more than one surface component enables Cbp1 *B. fragilis* to bind to collagen. This is not altogether unexpected as adhesion to ECM components is often achieved by the contribution of several cell surface components. It is interesting to note that Cbp1 was associated with the outer membrane of the bacterium, and adhesion of *B. fragilis* to host cells has been associated with outer membrane vesicles and surface polysaccharides [36]. Future work should not only aim to identify the other collagen binding proteins already observed in this study, but also investigate the role of the extracellular and cell associated components mentioned above, together with other structures known to be involved with bacterial adhesion, such as fimbriae [7]. Furthermore it would be of great interest to investigate the relationship of Cbp1 and *B. fragilis* cells in general to collagen proteins other than Type I collagen.

## Conclusion

The increasing incidence of antibiotic resistance in the important human opportunistic pathogen, *B. fragilis*, has prompted the search for new therapeutic targets such as virulence factors [6]. In this study, a **c**ollagen **b**inding glyco**p**rotein, designated Cbp1, was identified. Its function was confirmed by the loss of the relevant *B. fragilis* collagen adhesin bands/spots following insertional inactivation of the *cbp1* gene in the clinical isolate *B. fragilis* GSH18, as well as by the ability of purified recombinant Cbp1^His^ to bind collagen. This enabled the assignment of function as an adhesin to the ORF BF0586 in the type strain NCTC9343.

A pure substrate based *in vitro* binding system indicated redundancy with respect to collagen binding, as the *cbp1^−^* mutant continued to demonstrate significant collagen binding. Further research into the contribution of Cbp1 to the ability of the pathogen to bind and invade host cells *in vitro*, as well as *in vivo* assessment of virulence and immune response, will determine the functional role of this novel adhesin.
